# Potential Physiological Relevance of ERAD to the Biosynthesis of GPI-Anchored Proteins in Yeast

**DOI:** 10.3390/ijms22031061

**Published:** 2021-01-21

**Authors:** Kunio Nakatsukasa

**Affiliations:** Graduate School of Science, Nagoya City University, Yamanohata 1, Mizuho-cho, Mizuho-ku, Nagoya, Aichi 467-8501, Japan; nakatsukasa@nsc.nagoya-cu.ac.jp

**Keywords:** ERAD, GPI-anchored protein, Hrd1, Doa10, Ubc6, Ubc7, Ca^2+^/Mn^2+^ P-type ATPase, *Saccharomyces cerevisiae*

## Abstract

Misfolded and/or unassembled secretory and membrane proteins in the endoplasmic reticulum (ER) may be retro-translocated into the cytoplasm, where they undergo ER-associated degradation, or ERAD. The mechanisms by which misfolded proteins are recognized and degraded through this pathway have been studied extensively; however, our understanding of the physiological role of ERAD remains limited. This review describes the biosynthesis and quality control of glycosylphosphatidylinositol (GPI)-anchored proteins and briefly summarizes the relevance of ERAD to these processes. While recent studies suggest that ERAD functions as a fail-safe mechanism for the degradation of misfolded GPI-anchored proteins, several pieces of evidence suggest an intimate interaction between ERAD and the biosynthesis of GPI-anchored proteins.

## 1. Introduction

Secretory and membrane proteins are translocated to the endoplasmic reticulum (ER), where they are folded into a three-dimensional conformation. In the ER, molecular chaperones recognize unfolded polypeptides and facilitate their folding by binding to amino acid patches containing exposed hydrophobic side chains [1]. Proteins that acquire the correct conformation are transported from the ER to the Golgi apparatus. However, despite such protection, the maturation of proteins is an inherently error-prone process, and misfolded proteins are frequently generated. To remove the potentially toxic misfolded proteins, eukaryotes have evolved two systems. The first system is the unfolded protein response (UPR), which involves the upregulation of the factors that increase the protein-folding capacity of the ER. The UPR also facilitates the transport of misfolded proteins to the lysosome/vacuole, where they are degraded. The stress conditions caused by the accumulation of misfolded proteins (ER stress) induce membrane expansion of the ER to alleviate the stress independently of an increase in ER chaperone levels [2,3]. The second system is ER-associated degradation (ERAD), by which terminally misfolded proteins are specifically recognized, retained in the ER, and retro-translocated to the cytoplasm, where they are ubiquitinated and degraded by the proteasome. Components of the ERAD machinery can also be induced by the UPR [4,5,6]. Thus, the UPR and ERAD constitute two arms of the ER quality control apparatus and play critical roles in maintaining ER homeostasis [6,7,8,9,10,11,12].

Various “model misfolded proteins” have been developed and used for the analysis of degradation pathways [13,14]. However, emerging evidence indicates that ERAD not only mediates the elimination of structurally abnormal proteins in the ER, but also contributes to the regulation of native proteins [15]. For example, ERAD targets properly folded proteins to regulate metabolic enzymes, transcription factors, and metal transporters at the plasma membrane [16,17,18,19]. To further elucidate the physiological roles of ERAD, it is imperative to identify native substrates. In addition, yeast-based genetic interaction studies may help discover novel associations between ERAD and other biological phenomena. This review describes the ERAD machineries in yeast and the findings of recent studies analyzing the biogenesis and quality control of misfolded glycosylphosphatidylinositol (GPI)-anchored proteins. The potential involvement of ERAD in manganese homeostasis, which might link the biogenesis of GPI-anchored proteins to ERAD, is also discussed.

## 2. ER-Associated Degradation in Yeast

### 2.1. The Hrd1 Pathway

**Figure 1:**
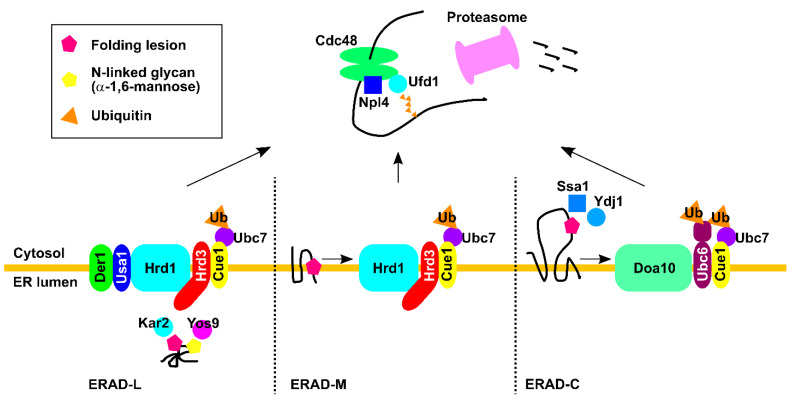
The ERAD (ER-associated degradation) pathway in *Saccharomyces cerevisiae* (see text for detail).

In yeast (*S. cerevisiae*), two dedicated ER membrane-associated E3 ligase complexes are involved in the recognition and degradation of misfolded proteins in the ER. The Hrd1 E3 ligase complex recognizes and targets misfolded luminal proteins, as well as ER membrane proteins with lesions at the transmembrane domain, for ubiquitination and degradation (Figure 1). These pathways are the so-called ERAD-L and -M pathways, respectively [17,20,21,22,23,24,25,26,27]. The Hrd1 complex consists of Hrd3, Usa1, Der1, Dfm1, Yos9, Kar2, Ubc7, and Cue1. Sucrose density gradient and systematic immunopurification analyses showed that Hrd1, Hrd3, Usa1, Der1, and Yos9 comprise the core complex [25,28]. During ERAD, a misfolded substrate is first recognized by several factors, including Yos9 (luminal lectin), Der1 (transmembrane protein), Kar2 (Hsp70 chaperone), or directly by Hrd1. The recognition mechanism for misfolded luminal glycoproteins has been studied extensively. The N-linked glycan is trimmed by glycosidase, and the terminal α1,6-mannose residue is recognized by the mannose 6-phosphate receptor homology (MRH) domain of Yos9 [29,30,31,32,33]. Non-glycosylated misfolded substrates may be recognized by Kar2. Both Yos9 and Kar2 bind to the luminal domain of Hrd3. In addition, unfolded and extended polypeptide segments may be recognized by the luminal domain of Hrd3 [34]. Until recently, differentiating the function of Hrd3 from Hrd1 stabilization was difficult because depletion of Hrd3 results in Hrd1 instability. However, removal of the ubiquitin-like domain of Usa1 caused Hrd1 to remain stable. This method was used to demonstrate that Hrd3 plays a direct role in facilitating the transfer of ubiquitin to substrates [35]. Usa1 mediates the interaction of Hrd1 with Der1, which is an inactive form of rhomboid protease, and transports substrates from the ER lumen to Hrd1 for degradation. Usa1 can also mediate the formation of an Hrd1 oligomer, which is critical for the degradation of ERAD-M substrates [21,27,36]. After recognition, the substrate is transferred to the Hrd1 complex for polyubiquitination. The transmembrane protein Cue1 is an ER membrane protein that recruits the E2 ubiquitin-conjugating enzyme Ubc7 to the Hrd1 complex [37]. Hrd1 also recruits the ER membrane protein Ubx2 to the complex, which anchors the AAA+ ATPase Cdc48/p97 to the membrane [38,39]. Then, Cdc48/p97 hydrolyzes ATP and liberates the substrate from the ER. Inactivation of Cdc48 leads to a formation of stalled retro-translocation complex containing Hrd1, Usa1, Hrd3, Der1, the 26S proteasome, Yos9, ubiquitinated substrates, and Cdc48. This suggests that substrate recognition and retro-translocation might be coupled, at least for some substrates [40]. Recognition of the integral membrane ERAD-M substrate is mediated by the transmembrane domain of Hrd1 [41] (Figure 1). The membrane substrates may exit the ER through a distinct pathway mediated by the Dfm1 rhomboid protein, which can also recruit the AAA+ ATPase Cdc48/p97 to the membrane for retro-translocation. Upon deletion of the *DFM1* gene, Hrd1 levels increase and the Hrd1 complex may be remodeled, thereby enabling a novel route of membrane protein retro-translocation. This supports the functional flexibility of the Hrd1 complex in response to ER stress [42,43].

### 2.2. The Doa10 Pathway

Another E3 ligase complex involved in yeast ERAD is Doa10, which resides in the nuclear membrane and ER membrane. Doa10 is a 150 kDa protein with 14 transmembrane segments [44] that targets transmembrane substrates with cytoplasmic lesions for ubiquitination and degradation, a pathway called ERAD-C (Figure 1). Substrates with abnormal structural domains in multiple regions may be degraded depending on both the Hrd1 and Doa10 complexes [45,46]. Substrates of the Doa10 complex include single- or multi-spanning membrane proteins in the ER and the inner nuclear membrane, as well as soluble proteins in the cytosol and nucleoplasm [19,44,47,48,49,50,51,52]. Unlike the Hrd1 complex, which comprises multiple components, the Doa10 complex is relatively simple and contains three ubiquitination enzymes: Ubc6, Ubc7, and Cue1. However, in contrast to the Hrd1 pathway, the mechanism by which the Doa10 complex recognizes misfolded substrates is not well understood. Cytosolic chaperones, such as the Hsp70 Ssa1 and the cytosolic Hsp40s Ydj1 and Hlj1, facilitate substrate recognition [53,54,55]. The degrons of Doa10 substrates can be cytoplasmic [49,50] or located within the TM region [51]. During ubiquitination, Ubc6 attaches the first ubiquitin to a substrate, and Ubc7 extends ubiquitin chains using mostly lysine 48 linkages [56]. Ubiquitinated substrates are then retro-translocated to the cytosol by Cdc48/p97, which is recruited to the ER membrane by Ubx2 and/or Dfm1 [43,53,57]. The mechanism underlying the retro-translocation of membrane substrates, including Doa10 substrates, is ill-defined. The extraction of Ubc6, which is degraded in a Doa10-dependent manner [50,58], was recently reconstituted in vitro [52]. The results of this experiment suggest that the luminal domain is unfolded by the action of Cdc48/p97 in the cytosol and crosses the membrane in an unfolded state.

## 3. GPI-Anchored Proteins

**Figure 2:**
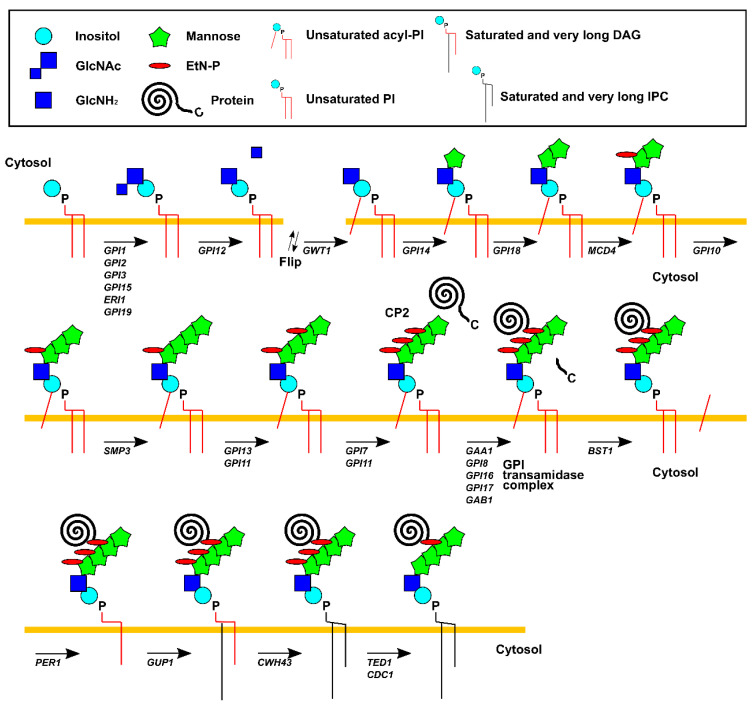
The pathway of GPI (glycosylphosphatidylinositol) biosynthesis in *Saccharomyces cerevisiae* (see text for detail).

GPI anchors are structurally complex glycophospholipids that are post-translationally attached to the C-terminus of secretory proteins (Figure 2). The highly conserved core structure of the GPI anchor precursor (CP2: complete precursor 2), which accumulates in the GPI transamidase mutant, comprises four mannoses (Man1, Man2, Man3, and Man4), three ethanolamine phosphate (EtN-P) substituents on Man1, Man2, and Man3, one acyl-phosphatidylinositol (acyl-PI), and one glucosamine (GlcN) [59]. More than 20 genes encoding enzymes involved in the biosynthesis of GPI anchors have been identified by genetic screening to isolate mutant cells that lack GPI proteins at the surface. GPI anchor synthesis begins on the cytosolic side of the ER membrane. The first step in GPI biosynthesis is the addition of *N*-Acetylglucosamine (GlcNAc) to phosphatidylinositol (PI). This transfer reaction is catalyzed by GPI-GlcNAc transferase, which consists of Gpi15, Eri1, Gpi3, Gpi19, Gpi2, and Gpi1 [60,61,62]. Next, the acetyl group of GlcNAc is removed by Gpi12, a GlcNAc-PI de-*N*-acetylase, to generate GlcN-PI, which is translocated by flippase to the luminal side of the ER membrane. Inside the ER, the acyltransferase Gwt1 adds an acyl chain, which is mostly palmitate, to the 2-position of the inositol in GlcN-PI, generating GlcN-(acyl) PI [63]. Subsequently, Man1 and Man2 are transferred from dolicholphosphomannose (Dol-P-Man) to GlcN-(acyl) PI by GPI mannosyl-transferase 1 (Gpi14) and GPI mannosyl-transferase 2 (Gpi18), respectively [64,65]. In addition, EtN-P is transferred from phosphatidylethanolamine (PE) to Man1 by EtN-P transferase (Mcd4) [66]. Then, Man3 is added to Man2 by GPI mannosyl-transferase 3 (Gpi10), and Man4 is added to Man3 by Smp3 before the addition of EtN-P to Man3 by EtN-P transferase 3 [67,68,69]. This complex comprises Gpi13, which is a catalytic subunit, and the stabilizing subunit Gpi11. Finally, EtN-P is added to Man2 by EtN-P transferase enzyme 2, which consists of the catalytic subunit Gpi7 and the stabilizing subunit Gpi11 [70,71].

Precursors of GPI-anchored proteins have a signal for GPI anchoring at the C-terminus and a conventional signal sequence for ER translocation at the N-terminus. The GPI anchoring sequence contains a C-terminal hydrophobic domain that is separated from the upstream GPI-attachment site (the “ω site”) by a short stretch of hydrophilic amino acids [72]. Soon after translation of the precursor protein by ribosomes and its translocation into the ER membrane are completed, the C-terminus of the protein is conjugated to the amine group of EtN-P by the GPI transamidase complex, which consists of five essential proteins: Gpi8, Gpi17, Gpi16, Gab1, and Gaa1 [73,74,75,76,77]. Of these, Gpi8, a catalytic subunit that is homologous to caspase-like cysteine proteases, cleaves the C-terminus of substrate proteins. This reaction is a prerequisite for the transamidation reaction [78,79,80]. After attachment, the GPI anchor is subject to a sequence of remodeling reactions on both the lipid and sugar moieties. These reactions occur exclusively inside the ER and are catalyzed by Bst1, Per1, Gup1, and Cwh43 in yeast [81,82,83,84]. Bst1 is a phosphatidylinositol deacylase that mediates inositol deacylation. This step is required for downstream lipid remodeling. Per1 removes the unsaturated fatty acid at the sn2 position, and Gup1 adds C26 fatty acids. Cwh43 is responsible for replacing diacylglycerol (DAG) with ceramide, which is a major lipid (saturated and very long inositolphosphoceramide) component of mature GPI anchors in yeast. Subsequently, Cdc1 and Ted1 remove the EtN-P of the first and second mannose, respectively [85,86]. These two enzymes are homologs of mammalian PGAP5, a membrane-spanning enzyme that possesses a metal-containing phosphoesterase motif in the luminal domain and removes EtN-P from the second mannose in the ER [87]. The removal of EtN-P from the second mannose is a prerequisite for the recognition of GPI-anchored proteins by the p24 complex and their exit from the ER. The localization of Cdc1 to the cis/medial Golgi apparatus, and not to the ER, was demonstrated recently. Removal of EtN-P from the second mannose by Ted1 in the ER and from the first mannose by Cdc1 in the Golgi apparatus may serve as a quality assurance signal for GPI-anchored proteins.

## 4. Quality Control of GPI-Anchored Proteins

In yeast, a mutant version of Gas1, β-1,3-glucanosyltransferase, which is referred to as Gas1*, is a well-studied model substrate for the quality control of misfolded GPI-anchored proteins. Gas1* contains a point mutation (G291R) that renders the protein misfolded and leads to its degradation [88,89]. Accumulating evidence suggests that Gas1* is not an efficient ERAD substrate. Only a small fraction is delivered to the ERAD pathway, while the vast majority of proteins escape Hrd1-dependent degradation; however, they are rapidly recognized by the p24 complex, including Emp24, exported from the ER, and delivered to the vacuole for degradation. p24 family proteins are conserved transmembrane proteins of ~24 kDa that function as cargo receptors for GPI-anchored proteins. The budding yeast p24 family is composed of three p24α (Erp1, Erp5, Erp6), one p24β (Emp24), three p24γ (Erp2, Erp3, Erp4), and one p24δ (Erv25) [90]. These are single-transmembrane proteins with a short (10–20 amino acids) C-terminal cytoplasmic tail. The cytoplasmic tail can interact with both COPI (Coat Protein I) and COPII (Coat Protein II) subunits. The luminal portion of these proteins may contribute to the formation of p24 oligomer and also participate in the cargo recognition. In mammalian cells, the misfolded version of the prion protein (PrP*), which is a GPI-anchored protein, is not degraded by ERAD but rather exported from the ER despite the misfolding [91]. PrP* is recognized by the p24 complex and delivered to the plasma membrane, from where it is transported to the lysosome for degradation [92]. When the ER is loaded with high amounts of misfolded proteins and its capacity is saturated during ER stress, misfolded PrP* dissociates from resident ER chaperones and is rapidly released into the secretory pathway in a process called rapid ER stress-induced export (RESET) [92]. This response is faster than the activation of the UPR and reduces the load of aberrant proteins in the ER, thereby maintaining protein homeostasis in the ER.

Then, what is the difference between misfolded GPI-anchored proteins and general ERAD substrates? When GPI anchor attachment is impaired, the misfolded protein moiety, which is free from the ER membrane, is rarely targeted to the vacuole. Instead, a considerable portion of these proteins is delivered to the ERAD pathway. The protein moiety of Gas1* is delivered to the Hrd1-dependent degradation pathway. In mammals, when GPI anchor attachment is prevented, PrP* is targeted for ERAD. Therefore, the protein moiety of the misfolded GPI-anchored proteins can be disposed by ERAD. It is possible that the GPI anchor sterically obstructs step(s) during ERAD, including retro-translocation and/or other steps. However, when GPI anchor remodeling proceeds incorrectly in cells lacking Bst1, Cwh43, or Ted1, GPI-anchored proteins are not efficiently exported from the ER; instead, they are retained in the ER and delivered to the Hrd1-dependent ERAD pathway [93]. This observation supports the idea that a GPI anchor does not pose a steric obstruction to the ERAD of misfolded GPI-anchored proteins. The ER retention time of misfolded GPI-anchored proteins may be determined by the remodeling status of the GPI anchor, which is directly coupled to ER export. To ensure the correct sequential synthesis of GPI-anchored proteins, it would be beneficial to remove those possessing an immature GPI anchor from the ER. Thus, the quality control of GPI-anchored proteins may be more severe than that of normal secretory proteins. This idea is further supported by the recent observation that Cdc1, a remodeling enzyme for GPI-anchored proteins, resides in the *cis*/medial Golgi apparatus, where additional quality control systems might be required [94]. In addition, a recent study showed that PrP* is trafficked from the ER to lysosomes in a complex with ER-derived chaperones, including calnexin and cargo receptors [95]. These interaction partners are critical for rapid endocytosis. Resident ER factors not only protect misfolded GPI-anchored proteins from aggregation during trafficking, but also ensure that they are subject to quality control at the plasma membrane and endocytosis to lysosomes.

## 5. Potential Physiological Relevance of ERAD to the Biosynthesis of GPI-Anchored Proteins

As mentioned above, emerging evidence suggests that ERAD functions as a fail-safe mechanism for the degradation of misfolded GPI-anchored proteins when the vacuole/lysosomal route is impaired. However, several observations support the physiological relevance of ERAD to the biosynthesis of GPI-anchored proteins.

### 5.1. Genetic Interactions between GPI and ERAD Genes

Systematic studies in yeast have suggested several positive and negative genetic interactions between genes encoding ERAD components and GPI biosynthetic factors [96]. Negative genetic interactions refer to double mutants that exhibit a more severe fitness defect than expected [97]. Conversely, positive genetic interactions refer to double mutants with a less severe growth defect than anticipated. These genes may contain genes encoding components of the same nonessential protein complex [97]. Genes required for relatively the later steps of GPI biosynthesis tend to interact with ERAD genes. For example, *HRD1* shows positive genetic interactions with *GPI8*, *GPI10*, *GPI11*, and *GPI17*. Similarly, *HRD3* shows positive genetic interactions with *GPI8*, *GPI10*, and *GPI17*, and negative genetic interactions with *GPI19*. *UBC7* shows positive genetic interactions with *GPI13*, *GPI16*, and *GPI17* [96]. Other interactions between major ERAD components and factors involved in the biosynthesis of GPI-anchored proteins are listed in Table 1. Although the reasons for these genetic interactions are currently unknown, one possible explanation is that endogenous substrates that accumulate upon ERAD deficiency positively or negatively affect the GPI deletion phenotype.

In mammals, genome-wide CRISPR-Cas9 (Clustered Regularly Interspaced Short Palindromic Repeats/CRISPR-Associated Proteins 9) genetic screening suggests that disruption of *HRD1* or several other ERAD components enhances GPI synthesis in GPI-transamidase-deficient cells [98]. The proposed scenario is that cells use ERAD to suppress GPI synthesis by degrading unknown protein factor(s) or endogenous substrate(s) that normally enhance the biosynthesis of GPI. Disruption of ERAD may cause the accumulation of such factor(s), which can lead to increased free GPIs including its biosynthetic intermediates as well as mature forms in GPI-transamidase-deficient cells [98].

### 5.2. Quality Control of Proteins that Harbor the GPI Anchoring Signal in the Cytosol

Molecular recognition events, including protein targeting to the organelle, are inherently imperfect because of intrinsic limits on specific binding. Indeed, during protein targeting to the ER, many secretory proteins fail to associate with the signal recognition particle (SRP) and can be detected in the cytosol before their translocation [99,100]. Under normal conditions in mammalian cells, the efficiency of ER translocation is not high: it may range from 60% to 95% [101]. This implies that the cell must be equipped with a quality control system to monitor a significant number of un-translocated proteins in the cytosol. The existence of a surveillance system that targets SRP-independent substrates for degradation is also likely. These would include a precursor form of GPI-anchored proteins whose C-terminal signal is hydrophobic. In yeast, GPI-anchored proteins that are not translocated to the ER are degraded on the cytosolic face of the ER. This degradation pathway is termed prERAD (Pre-insertional ERAD) and relies on opposing forces of the ubiquitin ligase Doa10 and the deubiquitinating enzyme Ubp1 [102].

### 5.3. Exit of GPI-Anchored Proteins from the ER Is Affected by the Perturbation of Manganese Homeostasis

Studies of mammalian cells show that PGAP5, an ER membrane protein, has a metal-containing phosphate esterase motif in the lumen and requires manganese ions (Mn^2+^) for its enzymatic activity [87]. PGAP5 catalyzes the removal of the second EtN-P after GPI is transferred to the protein. The removal of EtN-P from GPI-glycan by PGAP5 is required for the efficient transport of GPI-anchored proteins from the ER to the Golgi apparatus. As mentioned above, *S. cerevisiae* has two putative homologs of PGAP5, Cdc1 and Ted1. Cdc1 is a Mn^2+^-dependent enzyme that interacts with genes involved in GPI fatty acid remodeling [85,103]. Cdc1 was recently shown to have mannose-ethanolamine phosphate phosphodiesterase activity, and it is responsible for the removal of EtN-P from Man1 [85]. Transport of Gas1 from the ER is delayed in *ted1*∆ cells [104,105], although it is not currently clear whether Ted1 is a manganese-dependent enzyme. Perturbation of manganese homeostasis by depletion of Spf1, a P5-type ATPase that regulates manganese transport into the ER, causes the defective export of GPI-anchored proteins from the ER, and their accumulation in the ER [106]. These observations in mammals and yeast suggest that manganese homeostasis is critical for the biosynthesis of GPI-anchored proteins.

### 5.4. Possible Involvement of ERAD in the Maintenance of Manganese Homeostasis

I found a genetic interaction between ERAD and Pmr1, a P-type Ca^2+^- and Mn^2+^-transporting ATPase that is localized in the Golgi membrane [107], in yeast. Pmr1 is a prototypic member of the Ca2^+^-ATPase family of transporting ATPases, which are found in a variety of organisms, including fungi, *Caenorhabditis elegans*, *Drosophila melanogaster*, and mammals [108,109,110]. Both the calcium and manganese ions that are transported by Pmr1 into the Golgi lumen enable proper processing and trafficking of proteins through the secretory pathway [107,111,112,113]. While calcium is mainly required for protein trafficking [114], manganese is an essential enzymatic co-factor for glycosyltransferases that catalyze protein glycosylation in the secretory pathway [112,115]. Pmr1 transports excess cytosolic manganese into the Golgi lumen and mediates its export from the cell via secretory pathway vesicles. Thus, Pmr1 contributes to the cellular detoxification of manganese [115]. Yeast cells with a deleted *PMR1* gene (*pmr1*∆ cells) show a pleiotropic phenotype. For example, cells lacking Pmr1 are sensitive to ethylene glycol-bis(β-aminoethyl ether)-N,N,N′,N′-tetraacetic acid (EGTA), an effective chelator of calcium, manganese, and other divalent metal ions [107]. Depletion of Pmr1 results in the accumulation of manganese and calcium in the cytoplasm as well as depletion of these ions from the Golgi apparatus. Moreover, cells lacking Pmr1 display a defect in carboxy peptidase Y (CPY) trafficking [112] and a defect in the degradation of CPY*, a typical ERAD-L substrate [116]. Defects in the human ortholog of *PMR1*, ATP2C1, are associated with Hailey–Hailey disease, an autosomal dominant blistering skin disorder [108,117].

**Figure 3:**
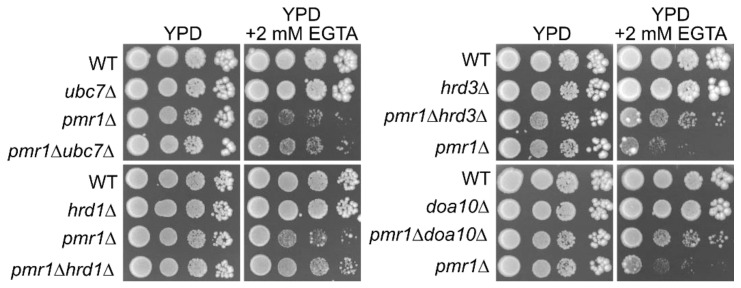
Deletion of major ERAD components suppresses the EGTA (Ethylene glycol-bis(β-aminoethyl ether)-N,N,N′,N′-tetra-acetic acid) sensitivity of *pmr1*∆ cells. Serial 10-fold dilutions of yeast cultures were spotted onto yeast extract-peptone-dextrose medium (YPD) or YPD containing 2 mM EGTA. Plates were incubated for 3–7 days. The strains used here were BY4741 (wild-type) and its mutant derivatives.

A previous large-scale analysis suggested a genetic interaction between *PMR1* and *UBC7* [118]. To confirm this observation, I constructed *ubc7*∆, *pmr1*∆, and *ubc7*∆*pmr1*∆ mutant strains and analyzed their growth (Supplementary Materials). However, double mutant cells grew normally compared with isogenic wild-type strains or single mutant strains, at least on yeast extract-peptone-dextrose (YPD) medium (Figure 3). Next, I tested the EGTA sensitivity of these strains because *pmr1*∆ cells were previously shown to be sensitive to EGTA. Consistently, *pmr1*∆ cells were sensitive to EGTA, whereas cells with the deleted *UBC7* gene grew similarly to wild-type cells (Figure 3). Interestingly, I found that the deletion of *UBC7* suppressed the EGTA sensitivity of *pmr1*∆ cells, indicating that the deletion of *UBC7* suppresses the EGTA sensitivity of *pmr1*∆ cells. To further confirm this result, I analyzed the genetic interactions between *PMR1* and other ERAD components. As shown in Figure 3, the EGTA sensitivity of *pmr1*∆ cells was rescued by the depletion of Hrd1, a retro-translocation channel for luminal ERAD substrates. A similar trend was observed for Hrd3, a component of the Hrd1 complex, and for Doa10, a membrane E3 ligase that targets misfolded membrane proteins with cytoplasmic lesions. These results suggest a potential physiological role of ERAD in maintaining calcium and manganese homeostasis in the Golgi apparatus.

The mechanism by which depletion of ERAD might rescue the EGTA-sensitive phenotype of *pmr1*∆ is currently unclear. One possible scenario is that a native luminal and/or membrane protein(s) accumulates upon ERAD deficiency and affects the *pmr1*∆ phenotype. Furthermore, there may be many kinds of substrates that accumulate in ERAD-defective cells and affect the phenotype of *pmr1*∆ cells. One candidate is Cdc1, which suppresses the EGTA sensitivity of *pmr1*∆ cells when overexpressed [85,119,120,121]. Consistently, N-terminal hemagglutinin-tagged Cdc1 is stabilized in the Hrd3 mutant [85]. However, a recent study showed that Cdc1 localizes to *cis* and medial Golgi when tagged with the fluorescent mNeon protein at its N- or C-terminus [94], although a previous study showed that Cdc1 localizes to the ER when tagged with the myc-epitope at the N-terminus [120]. The reason for these discrepancies is not clear; however, the removal of EtN-P from Man 1 would be a critical step for the quality control of GPI-anchored proteins before they are delivered to the plasma membrane. Nonetheless, it will be essential to test the localization and stability of a purely endogenous untagged version of Cdc1 to fully understand the observed phenomena.

## 6. Conclusions

Unidentified endogenous ERAD substrates are key to understanding the potential relationship between ERAD and the biosynthesis of GPI-anchored proteins. The simple genetic interaction reported by systematic studies implies the existence of an endogenous ERAD substrate that may affect the phenotype of cells defective in GPI biosynthesis. Mammals may express a putative positive regulator of GPI biosynthesis whose stability is regulated by ERAD. The rescue of the EGTA sensitivity of *pmr1*∆ cells by deletion of ERAD components could also be explained by the accumulation of endogenous substrates. This suggests that ERAD could control manganese homeostasis, which is critical for the ER exit of GPI-anchored proteins. Current data indicate that the role of ERAD is not limited to the degradation of misfolded proteins and may play a critical role in the regulation of cellular phenomena, even under normal growth conditions. The identification of an endogenous substrate of Hrd1, Doa10, as well as the dedicated ubiquitin ligases in mammals [15], would be important to fully understand the physiological roles of ERAD.

## Figures and Tables

**Table 1:** Positive and negative genetic interactions between genes encoding ERAD components and GPI biosynthetic factors. N: negative genetic interactions; P: positive genetic interactions.

	*GPI2*	*GPI8*	*GPI10*	*GPI11*	*GPI13*	*GPI16*	*GPI17*	*GPI19*
*HRD1*		P	P	P			P	
*HRD3*		P	P				P	N
*UBC7*					P	P	P	
*USA1*		P		P	P		P	
*DER1*	N			P	P			
*YOS9*					P		N	N
*DOA10*				P				

## Data Availability

Data is contained within the article or supplementary material.

## Supplementary Materials

Supplementary materials can be found at https://www.mdpi.com/1422-0067/22/3/1061/s1.

## Abbreviations

ERAD	Endoplasmic reticulum-associated degradation
EGTA	Ethylene glycol-bis(β-aminoethyl ether)-*N*,*N*,*N*′,*N*′-tetra-acetic acid
GPI	Glycosylphosphatidylinositol
EtN-P	Ethanolamine phosphate
Man	Mannose
GlcN	Glucosamine
GlcNAc	*N*-Acetylglucosamine
UPR	Unfolded protein response
prERAD	Pre-insertional ERAD
