# Supplementary material for: Potential Physiological Relevance of ERAD to the Biosynthesis of GPI-Anchored Proteins in Yeast
Source: Int J Mol Sci. 2021 Jan 21;22(3):1061. doi: 10.3390/ijms22031061 (PMC7865462; doi:10.3390/ijms22031061)
Supplement: Supplementary file 1 [file ijms-22-01061-s001.pdf]

Materials and Methods

Strains and plasmids used in this study

The yeast strains (*S. cerevisiae*) used in this study are listed in Table 1. Standard genetic techniques were used to construct strains and plasmids. Cells were grown in YPD-rich medium (YPD+5xAde: 1% yeast extract, 1% peptone, 100 mg/L of adenine hydrochloride, and 2% glucose) in the presence or absence of indicated concentration of EGTA.

Table 1. Yeast strains used in this study.

Strain	Genotype	Source
BY4741	*MAT***a** *his3Δ1 leu2Δ0 met15Δ0 ura3Δ0*	Lab stock
SGA3	*MAT***a** *his3Δ1 leu2Δ0 met15Δ0 ura3Δ0 ubc7Δ::KanR*	Open biosystems
SGA41	*MAT***a** *his3Δ1 leu2Δ0 met15Δ0 ura3Δ0 hrd1Δ::KanR*	Open biosystems
SGA47	*MAT***a** *his3Δ1 leu2Δ0 met15Δ0 ura3Δ0 hrd3Δ::KanR*	Open biosystems
SGA56	*MAT***a** *his3Δ1 leu2Δ0 met15Δ0 ura3Δ0 doa10Δ::KanR*	Open biosystems
SGA284	*MAT***a** *his3Δ1 leu2Δ0 met15Δ0 ura3Δ0 pmr1Δ::natR*	This study
SGA285	*MAT***a** *his3Δ1 leu2Δ0 met15Δ0 ura3Δ0 ubc7Δ::KanR pmr1Δ::natR*	This study
SGA287	*MAT***a** *his3Δ1 leu2Δ0 met15Δ0 ura3Δ0 hrd1Δ::KanR pmr1Δ::natR*	This study
SGA288	*MAT***a** *his3Δ1 leu2Δ0 met15Δ0 ura3Δ0 hrd3Δ::KanR pmr1Δ::natR*	This study
SGA289	*MAT***a** *his3Δ1 leu2Δ0 met15Δ0 ura3Δ0 doa10Δ::KanR pmr1Δ::natR*	This study
